# Impacte des maladies immunitaires sur la grossesse expérience du Service de Gynécologie Obstétrique de l'hôpital Militaire Moulay Ismail

**DOI:** 10.11604/pamj.2016.24.38.8518

**Published:** 2016-05-10

**Authors:** Omar Laghzaoui

**Affiliations:** 1 Université Sidi Mohammed Ben Abdellah Faculté de Médecine et de Pharmacie, Fès, Maroc

**Keywords:** Lupus, Behçet, polyarthrite rhumatoïde, sclérose en plaque, sclérodermie, Lupus, Behçet, rheumatoid arthritis multiple, sclerosis, scleroderma, pregnancy complications

## Abstract

L'influence du statut hormonal au cours des maladies auto-immunes est clairement établie, avec une prévalence maximale pendant la période d'activité génitale d'où l'intérêt de notre étude rétrospective de 32 dossiers de patientes enceintes présentant des pathologies auto-immunes. Les rechutes de la maladie au cours de la grossesse sont surtout observées chez les gestantes présentant le Lupus érythémateux disséminé et la maladie de Behçet alors qu'en poste partum les complications sont observées en cas de polyarthrite rhumatoïde, sclérose en plaque et la sclérodermie. Les complications fœtales dépendent du stade et du type de la maladie auto immune ainsi que l'association à d'autres pathologies. La prise en charge multi disciplinaire et l'ajustement du traitement abouti à stabiliser la maladie auto immune et améliore le pronostique fœtale.

## Abstract (en)

The influence of hormonal status during the autoimmune disease is clearly established, with peak prevalence during the reproductive years where the interest of our retrospective study of 32 cases of pregnant patients with autoimmune pathologies. Relapses of the disease during pregnancy are especially observed in pregnant with lupus erythematosus and Behçet's disease while in post-partum complications were observed in cases of rheumatoid arthritis, multiple sclerosis and scleroderma. The fetal complications depend on the stage and type of autoimmune disease and the association with other pathologies. Taking multi-disciplinary charge and adjustment of treatment resulted in stabilizing the autoimmune disease and improves fetal prognosis.

## Introduction

La grossesse est un facteur de risque des complications chez la femme porteuse d'une maladie auto immune et ceci à travers les modifications hormonales. Cette étude rétrospective de 32 dossiers de patientes suivies au service de gynécologie-obstétrique de l′hôpital militaire Moulay Ismail de Meknès, pour maladie auto immune et grossesse a pour but de repérer ces complications fœto-maternelles et d’épier la vertu de la prise en charge.

## Méthodes

Cette étude rétrospective a été réalisée sur une période de cinq ans, de janvier 2009 à décembre 2014, au service de gynécologie-obstétrique de l′hôpital militaire Moulay Ismail Meknès Les critères d'inclusion sont cliniques, biologiques et radiologique des patientes enceinte connues porteuses de maladie auto immune ou ayant présentées la maladie au cours de la grossesse. L’étude des dossiers a pu collecter 32 patientes porteuses maladie auto immune et grossesse sur un total de 12040 dossiers de femmes enceinte: -Quinze dossiers de lupus et grossesse. -Huit dossiers de polyarthrites rhumatoïdes et grossesse. -Six dossiers de maladie de Behçet et grossesse. -Deux dossiers de scléroses en plaque et grossesse. -Un dossier de sclérodermie et grossesse.

## Résultats

La fréquence de l′association maladie auto immune et grossesse est de 0,26% dans notre série. L′âge moyen de nos parturientes est de 32 ans avec des extrêmes allant de 24 à 42 ans. Le taux moyen de gestation est de 2,86 gestes avec un maximum de 4 gestes et un minimum de 1 seul geste. Le taux moyen de parité de nos patientes est de 2,2 avec un nombre de multipares significativement supérieur. Concernant les antécédents Deux patientes sont suivies pour HTA sous traitement, deux patientes sont diabétiques, dont une est compliquée d′insuffisance rénale sous hémodialyse. L’étude des antécédents familiaux trouve un lupus familial chez deux patientes, une thyroïdite d'Hashimoto chez la mère d'une patiente et une maladie de Behçet familiale chez trois patientes.

**Figure 1:**
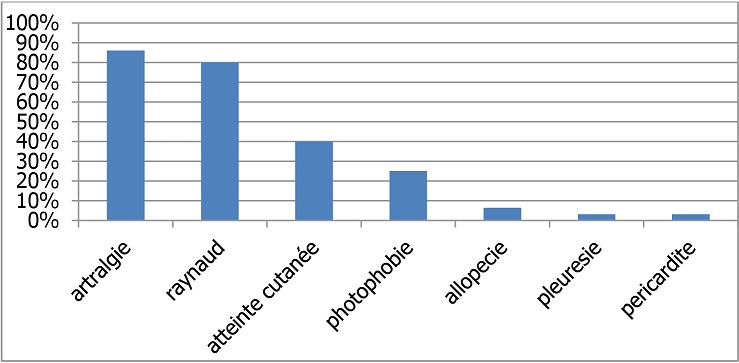
Manifestations cliniques liées au lupus chez nos patients

**Lupus érythémateux disséminé:** L'association la plus fréquemment retrouvée dans notre étude est Le lupus érythémateux disséminé et grossesse (46,8% cas). Les quinze patientes étaient connues porteuses du lupus avant la gestation avec des manifestations clinique représentées dans la Figure 1. Les anticorps antinucléaires et les anticorps anti DNA natifs étaient positifs chez toutes les patientes. Les anticorps anti phospholipides étaient positifs chez deux patientes. Le CH50 était diminué chez six patientes soit 40%, quatre d'entre elles avaient aussi une haptoglobine élevée soit 26,6%. Les anomalies de l′hémogramme étaient présentes chez neuf patientes ce qui représente une fréquence de 60%(L'anémie chez sept patientes et une leucopénie plus thrombopénie chez trois patientes). La protéinurie de 24h était positive chez deux patientes qui ont une insuffisance rénale, dont une est sous hémodialyse. L’évolution au cours de la grossesse était marquée par des poussées évolutives chez cinq patientes qui avaient arrêtées l'hydroxychloroquine (plaquenil). Une patiente qui avait développé une néphropathie lupique compliquée d'insuffisance rénale à diurèse conservée et une pleurésie lupique ce qui a suscité la reprise du cyclophosphamide. Une patiente ayant nécessitée une ponction péricardique pour tamponnade et traitement aux corticoïdes. Les trois autres patientes ont eu des polyarthralgies avec atteintes cutané jugulées par les corticoïdes.

**Figure 2:**
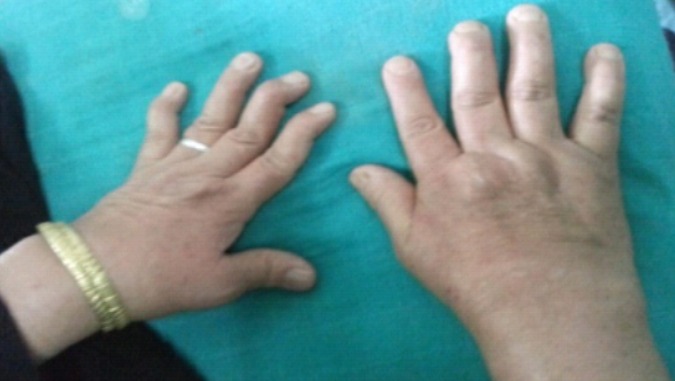
Déformation des phalanges en col de cygne PAR

**La polyarthrite rhumatoïde (PR):** Les huit patientes atteintes de polyarthrite rhumatoïde avaient un âge avancé entre 36 et 42 ans lors de leurs grossesses et étaient déjà au stade de ténosynovites avec déformations articulaires des phalanges (Figure 2), sur le plan biologique le syndrome inflammatoire ainsi que le facteur rhumatoïde étaient quasi constant chez toutes les patientes. L'imagerie radiologique des extrémités plaide en faveur de la polyarthrite rhumatoïde. Durant la période de gestation aucune patiente n'a eu de poussées évolutive bien au contraire trois d'entre elles ont eu une amélioration de la symptomatologie douloureuse avec une nette diminution de la vitesse de sédimentation. Par contre en postpartum on a soulevé une recrudescence du syndrome inflammatoire avec myasthénie chez cinq patientes, et un purpura thrombopénique chez une patiente chez qui on a arrêté l'allaitement pour donner le méthotrexate. Les autres patientes ont été mises sous anti-inflammatoires non stéroïdiens.

**Figure 3:**
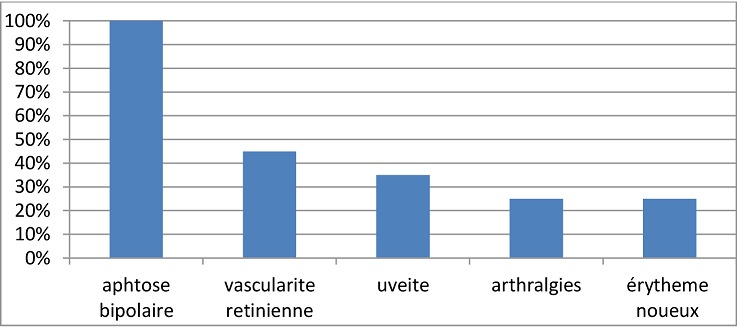
Manifestations cliniques chez les patientes portant la maladie de Behçet

**Figure 4:**
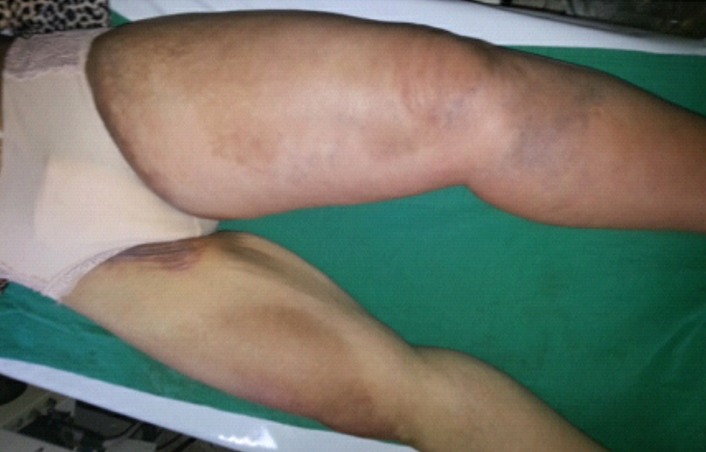
Phlébite du membre inferieur gauche sur maladie de Behçet

**La maladie de Behçet:** Les six patientes présentant la Maladie de Behçet avaient comme particularité un âge inférieur à 28 ans et trois d'entre elles sont des sœurs (maladie de Behçet familiale). Les manifestations cliniques relevées chez les patientes sont dominées par l'aphtose bipolaire (Figure 3). Les manifestations biologiques étaient marquées par la présence d'un syndrome inflammatoire avec élévation de la vitesse de sédimentation au cours des poussées de la maladie. Quatre patientes sur six ont présentées des poussées évolutives de la maladie, deux au cours de la grossesse et deux en post partum. Les complications relevées sont une péricardite jugulée par les corticoïdes, une uvéite postérieure avec baisse de l'acuité visuelle traitée par corticothérapie en collyre en association à des mydriatiques suivie d'injections péri-oculaires de corticoïdes, un cas de thrombose de la veine saphène interne en postpartum traitée par l'héparine à bas poids moléculaire (Figure 4), et une perforation hémorragique de l'intestin grêle en postpartum (forme appelée entéro-Behçet) traitée chirurgicalement. Les six patientes étaient sous colchicine avant et pendant la grossesse ainsi que des corticoïdes pendant les poussées.

**Figure 5:**
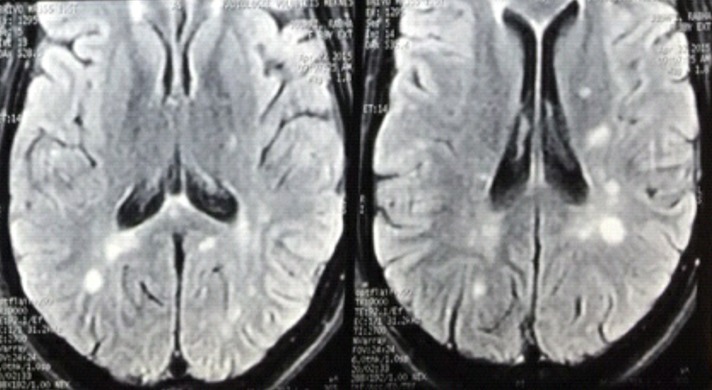
IRM cérébrale-images ovalaires péri ventriculaire (SEP)

**Figure 6:**
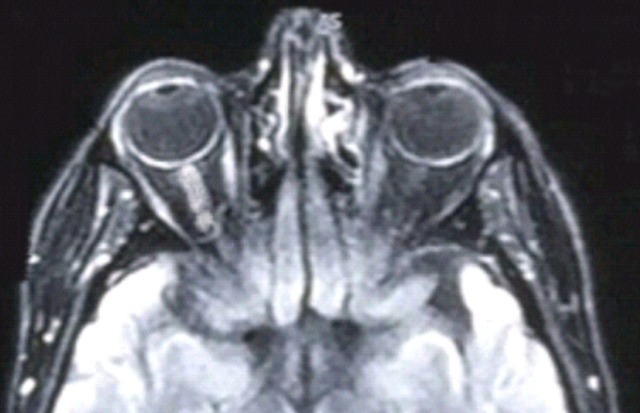
IRM névrite du nerf optique (SEP)

**Sclérose en plaque:** Les deux patientes suivies pour sclérose en plaque étaient stable sous corticoïdes avant la grossesse. La première patiente avait présenté une rechute de la maladie dans le postpartum immédiat par l'apparition d'une perte de sensibilité de la face interne du bras droit et fatigabilité à l′effort de celui-ci. L'IRM avait objectivé des lésions sous forme d′hyper signaux ovalaires de la substance blanche en péri ventriculaire (ventricules latéraux) sur les séquences T2 flair (Figure 5). La deuxième patiente a présenté deux mois après l'accouchement des céphalées en casque, avec vomissements, une importante faiblesse musculaire des membres supérieurs et une forte baisse de l'acuité visuelle avec au fond d’œil un œdème papillaire. L’étude biologique du liquide céphalorachidien a objectivée une proteinorrachie était à 0,26 g/l, la cellularité à 32 éléments/mm de globules blancs avec prédominance lymphocytaire, l’étude comparative de l’électrophorèse des protéines du LCR et celles du sérum avait trouvé un profil oligoclonal spécifique des IgG du LCR, affirmant la synthèse intrathécale d'IgG. L'IRM avait objectivé des lésions sous forme d′hyper signaux au niveau de la moelle cervicale et une névrite optique droite concluant à une rechute de la sclérose en plaque (Figure 6). Les deux patientes ont bénéficiées de bolus de corticoïdes puis le traitement par Inhibiteurs sélectifs des molécules d'adhésion avec arrêt de l'allaitement aboutissant à stabiliser la maladie.

**Figure 7:**
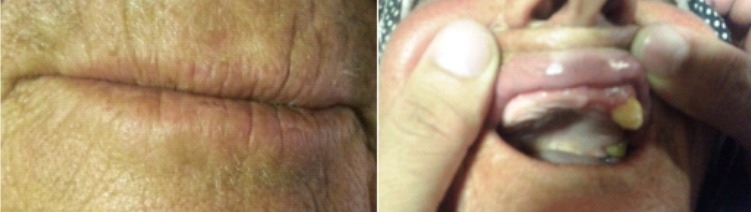
Sclérose labiale

**La sclérodermie:** La patiente atteinte de sclérodermie avait l’âge de 28 ans connue hypertendue et suivie pour diabète insulinodépendant. Elle a consulté au premier trimestre de la grossesse pour vomissements et une dysphagie aux solides accompagnés de phénomène de Raynaud, gonflement des mains avec ulcérations en regard des articulations, une perte de flexibilité secondaire au durcissement de la peau, en particulier sur les bras la bouche ainsi qu'une perte presque totale de la dentition (Figure 7). Le bilan biologique avait trouvé des anticorps antinucléaires et les anticorps anti centromères, dirigés contre les antigènes protéiques liés à l'ADN. La biopsie de la peau avait objectivée un infiltrat inflammatoire de cellules mononuclées de localisation péri vasculaire, associé à une augmentation du nombre et de l’épaisseur des fibres de collagène. Un traitement par les corticoïdes et inhibiteurs de la pompe à protons a pu stabiliser la maladie et améliorer la dysphagie. En postpartum la patiente a développé une néphropathie avec insuffisance rénale terminale ayant nécessité l'hémodialyse.

**Le suivie obstétrical:** L’âge gestationnel moyen de la première consultation est de 18 semaines d'aménorrhée avec des extrêmes de 08 à 26 SA. Aucune de nos patientes n'a bénéficié de consultation pré-conceptionnelle. L’évolution des grossesses étaient marquées par 2 cas de métrorragies un cas lié à un avortement en cours à 12SA et le deuxième un décollement minime de trophoblaste à 8 SA, les deux patientes étaient diabétiques en poussée lupique. Dix cas de retard de croissance intra-utérin chez cinq patientes qui ont présentées des poussées lupique. Quatre patientes suivies pour maladie de Behçet et La dernière patiente avait une sclérodermie. Trois patientes ont été admises pour menace d'accouchement prématuré sur poussées inflammatoires de leures maladies (deux lupus et une maladie de Behçet) une des patiente avait une poche des eaux rompue à 35SA, les deux autres à 32 et 34 SA à poche des eaux intacte, l’évolution s'est faite vers l'accouchement prématuré malgré la tocolyse (Tableau 1).

**Tableau 1:** Complications maternelles et fœtales au cours des maladies auto-immunes

	Complications maternelles		Complications de la grossesse	Complications fœtales
**Lupus**	Pendant la grossesse en postpartum			
	- 2 atteintes articulaires	-1 décès maternel éclampsie +Hellp syndrome	-1 AVS	-6 prématurés
	-1 insuffisance rénale	-	-5 RCIU	-1 MFIU
	-1 pleurésie	-	-2 MAP	-1mort fœtale en perpartum
	-1 péricardite	-	-1 HRP	-
**PAR**	-3 améliorations de la maladie	-5 poussées	-	-
	-	-1 purpura thrombopénique	-	-
	-	-	-	-
**Behçet**	-1Uvéite	-1Phlébite	-4 RCIU	
	-1Péricardite	-1Entéro- Behçet	-1 MAP	-3 prématurés
**SEP**	-	- 1névrite du nerf optique	-	-
	-	-1 myasthénie	-	-
**Sclérodermie**	-1dysphagie	-1 insuffisance rénale	-RCIU	-MFIU

-AVS : avortement spontané; -RCIU : retard de croissance in utéro;-MAP : menace d'accouchement prématuré; -HRP : hématome retro placentaire;-MFIU : mort fœtale in utéro

**L'issue des grossesses:** Dix-neuf grossesses ont étés menées à terme avec accouchement par voie basse soit 59,6% et neuf accouchements prématurés soit 41,4% (trois cas qui avaient échappées à la tocolyse et six cas après décision multidisciplinaire d'extraction fœtale par césariennes pour retard de croissance intra utérin avec stagnation du poids fœtal. Deux cas de mort fœtale in utero survenues à 30SA et à 31SA sur retard important de la croissance intra utérine sur insuffisance rénale. Un cas de mort per-partum à 30SA sur Placenta prævia plus hématome retro placentaire sur éclampsie et HELLP syndrome et un cas d'avortement à 12SA.

**Evolution maternelle:** Deux patientes ont présenté une pré-éclampsie sévère compliquée d’éclampsie, l'une d'elles est décédée de HELLP syndrome après un séjour de quinze jours en réanimation.

**Evolution f**œ**tale:** Le poids fœtal à la naissance variait entre 1750 et 3900 grammes, cinq nouveau-nés ont séjournés en couveuses pour prématurité, un nouveau-né traité par photothérapie pour ictère néo-natal, le bilan biologique n'a pas objectivé de lupus néo-natal.

## Discussion

Les maladies auto-immunes regroupent l'ensemble des pathologies ou l'auto anticorps est dirigé contre les constituants de n′importe quelle cellule de l'organisme, c'est donc une agression de l′organisme par son propre système immunitaire. La majorité de ces maladies se déclarent pendant la période d'activité génitale ce qui explique leurs liens avec la grossesse.

**Lupus érythémateux systémique:** Il s'agit d'une maladie caractérisée par la production d'anticorps antinucléaires dirigés en particulier contre l'ADN natif touchant surtout la femme en avec une incidence en France de 47 cas/100 000 l'année en 2010. Selon la littérature La grossesse constitue un facteur de complication du lupus érythémateux disséminé dans 60% des cas [1], dans notre étude le pourcentage est de 33,3%. Ces complications ne paraissent pas être lier ni aux nombre de grossesses ni à la durée de la rémission par contre un arrêt du traitement par l'hydroxychloroquine pourrait être la cause des rechutes, point qui a été soulevé dans notre étude. Les complications décrites qui augmente le plus la morbidité et mortalité maternelle sont l'atteinte rénale et cardiaque [1, 2]. Les complications obstétricales dues au lupus sont dominées par le retard de croissance intra-utérin et la prématurité dans 30% des cas, l'avortement ou mort fœtale in utero dans 20% des cas surtout en présence des anticorps anti phospholipides et/ou les anticorps anticardiolipine chez la mère et le bloc auriculo-ventriculaire complet dans 1,6% cas par passage trans-placentaire d'anticorps anti-SSA [3]. L'hydroxychloroquine a prouvé sa supériorité quant au traitement du lupus au cours de la grossesse, il ne présente pas d'effet toxique sur le fœtus bien au contraire limite les complications obstétricales et espace les rechutes. L'usage des corticoïdes par voie générale chez les patientes enceintes augmente le risque de développer le diabète l'hypertension artérielle et donc l’éclampsie ce qui diminue leurs utilisation [2, 3]. L'association aspirine et héparine parait comme le traitement de choix des avortements itératifs liés à la présence des anti-phospholipides. Ils agissent sur la synthèse de thromboxane plaquettaire et peuvent avoir un effet suppresseur sur l′anticoagulant lupique [4, 5]. Les facteurs de bon pronostic pour la mère et pour l'enfant sont la présence d'une maladie lupique quiescente pendant la grossesse, une pression artérielle bien contrôlée, une fonction rénale bien conservée et une protéinurie inférieure à trois gramme par jour [3, 5].

**La polyarthrite rhumatoïde:** La grossesse n'as pas d'impact négatif sur la patiente atteinte de polyarthrite rhumatoïde ainsi plus de 40% des femmes bénéficient d'une amélioration de leur symptomatologie pendant la grossesse selon la littérature [6]. Dans notre étude le pourcentage d'amélioration de la maladie est de 36,5%. En post-partum, la maladie redevient active entre 1 et 6 mois après l'accouchement avec un pic entre 6 et 12 semaines [6, 7]. L’élévation des concentrations d'estrogènes et de la progestérone pendant la grossesse agie sur l’élévation des cytokines anti inflammatoires (interleukine 1 récepteur soluble et TNFα récepteur soluble) ce qui explique l'amélioration de la polyarthrite rhumatoïde [6]. La possibilité d'existence concomitante du purpura thrombocytopénique et polyarthrite rhumatoïde peut augmenter la morbidité maternelle par le risque de saignement en per-partum et l′hémorragie cérébrale du fœtus pendant l′accouchement par passage trans-placentaire des anticorps anti-plaquettes provoquent une thrombocytopénie fœtale [7]. Les traitements utilisés pendant la gestation sont les anti-inflammatoires non stéroïdiens qui doivent être interrompue à 34 semaines d'aménorrhée pour éviter la fermeture précoce du canal artériel ce risque n'existe pas avec l'utilisation des anti-inflammatoires anti-cyclo-oxygénases (anti-COX B); les corticoïdes exposent aux risques d'HTA, diabète cortico-induit et la fente labiales à fortes doses [8]. Les médicaments comme le méthrotrexate, le chlorambucil, le cyclophosphamide, et l'anti-TNFα sont contrindiqués au cours de la grossesse et doivent être interrompus 3 à 6 mois avant d'envisager une grossesse [7, 8].

**La sclérose en plaque (SEP):** La SEP n'a pas d'influence sur la grossesse si ce n'est la spasticité des membres inferieures qui gêne l'accouchement par voie basse. La maladie évolue par poussées plus ou moins fréquentes et sévères, entre lesquelles les troubles régressent partiellement. Les symptômes varient en fonction des zones atteintes, l’évolution se fait vers une invalidité progressive. On a toutefois soulevé dans la littérature une atténuation des symptômes pendant la grossesse en particulier au troisième trimestre alors qu'il y a une aggravation de la maladie pendant les 3 premiers mois du post-partum c'est le cas de nos deux patientes. Cette évolution serait due à l'ostriol qui a une action anti-inflammatoire pour diminuer les rechutes pendant la grossesse, et la chute de cette hormone après l'accouchement laisse apparaitre les poussées évolutives [9, 10]. Selon des chercheurs à Emory University, la progestérone semble aussi protéger les neurones et favorise la remyélinisation des nerfs en augmentant le taux de la protéine de base constituant la myéline [11]. Des essais thérapeutiques multicentriques européens (L’étude POPART'MUS) sont en cours combinant progestérone par voie orale et œstradiol percutané pour atténuer les poussées du premier trimestre de grossesse et du post-partum chez les femmes atteintes de sclérose en plaque [10, 12]. La décision d'allaiter est un choix très personnel surtout qu'il est sans risque pour le nouveau-né, toute fois si l'indication se pose de reprendre un traitement immuno-actif l'allaitement sera suspendu. La programmation de la grossesse est prescrite par tous les auteures, envisager une grossesse après une période d'accalmie d'au moins un an, l'arrêt d'un traitement de fond (immunosuppresseurs: mitoxantrone, cyclophosphamide, méthotrexate et mycophénolate mofétil) six mois avant la conception [12].

**Maladie de Behçet:** La maladie de Behçet est une maladie auto-immune inflammatoire récidivante caractérisée par une vascularite multi systémique. La composante génétique de cette pathologie n'est pas clairement établie mais une forte association entre la pathologie de Behçet et le gène HLA-B51 a été observée, (55% des personnes au Japon atteintes présentaient le gène HLA-B51). La Turquie est le pays avec la prévalence la plus élevée au monde avec 110-420 cas/100 000 habitants, contre 2/100 000 en Angleterre et aux Etats-Unis [13]. Le diagnostic de la maladie est basé sur des arguments cliniques, étant donné qu'il n'y a aucun bilan biologique spécifique. Les manifestations cliniques sont une aphtose bipolaire, une atteinte inflammatoire oculaire, des lésions cutanées et une atteinte fréquente des articulations. Les atteintes du système nerveux central, des vaisseaux et du tractus gastro-intestinal constituent un stade avancé de la maladie. La formule sanguine montre fréquemment une anémie inflammatoire et une neutrophilie chez environ 15% des patientes. La vitesse de sédimentation et la C réactine protéine sont augmentés dans la phase active de la maladie [14–16]. Une recrudescence des symptômes de de la maladie de Behçet au cours de la grossesse ou le post-partum sont constatée dans (35,5%) dans la littérature, dans notre étude c’était (66,6%) toutes pendant la grossesse. Les manifestations les plus fréquentes lors de ces poussées sont l'aphtose bipolaire et les poussées inflammatoires oculaires, les plus graves sont les thromboses veineuses profondes. Les complications obstétricales sont d'une fréquence de 15,8% dominé par les fausses couches spontanées et il existe statistiquement une association significative entre les antécédents de thrombose veineuse profonde et le risque de complications obstétricales [14] L'azathioprine et interferon alpha ont fait leurs preuves entant que traitements des complications quel que soit le terme de la grossesse par contre l'azathioprine expose à un risque accru d'infection materno-fœtale (en particulier à cytomégalovirus) en raison de l'immunosuppression induite. Le traitement par la colchicine avant la conception semble diminuer La fréquence des rechutes pendant la grossesse [15].

**La sclérodermie:** La sclérodermie systémique est une maladie auto-immune qui touche plus particulièrement les femmes à un âge avancé ce qui explique la rareté des cas associé à la grossesse décrit dans la littérature. C'est une pathologie qui se caractérise par une sclérose progressive pouvant atteindre plusieurs organes et aboutir à une fibrose irréversible. Les signes cliniques sont dominés par un phénomène de Raynaud sévère et la fibrose cutanée. Les complications qui engagent le pronostic vital sont cardio-pulmonaires et rénales. La biopsie cutanée et la présence des anticorps antinucléaires spécifiques (anti-topoisomérase, anti-centromères, et anti-nucleolaire) confirment le diagnostic [17]. Les complications maternelles les plus fréquentes au cours de la grossesse sont l'aggravation du reflux gastro-œsophagien au premier trimestre, la crise rénale sclérodermique, similaire au cas de la patiente rapporté dans notre étude, et les poussées d'hypertension artérielle pulmonaire [18, 19]. Les complications obstétricales sont liées à la fibrose stromale et péri vasculaire du placenta ainsi qu’à l'existence d'anticorps anti-cardiolipines à l'origine de pré éclampsie et donc risque de retard de croissance intra-utérin, prématurité et mort fœtale in utero [17–19]. Les traitements de fond de la sclérodermie (méthotrexate, inhibiteurs des récepteurs de l'endothéline, d-pénicillamine et cyclophosphamide) sont tératogènes est donc contre indiqués au cours de la grossesse. Les inhibiteurs de la pompe à proton peuvent cependant être utilisés, pour le reflux gastro-œsophagien quel que soit le terme de la grossesse ainsi que les héparines de bas poids moléculaire et les anti-agrégants plaquettaires. Les inhibiteurs calciques et les analogues de la prostacycline sont utilisés pour traiter l'hypertension artérielle pulmonaire [19]. La grossesse est contre-indiquée en cas de fibrose pulmonaire importante, hypertension artérielle mal contrôlée associée ou non à un antécédent de crise rénale sclérodermique, hypertension artérielle pulmonaire élevée et si la fraction d’éjection du ventricule gauche est inférieure à 30% [17]. Le traitement de la sclérodermie reste encore un défi pour les médecins. Récemment, des inhibiteurs de kinase-ont montré un grand potentiel contre les maladies fibrotiques, ce qui pourrait être prometteur pour le traitement de la sclérodermie [20].

## Conclusion

Le diagnostic précoce des maladies auto immune au cours de la grossesse permet un meilleur control de la maladie faisant profiter la gestante des progrès considérables réalisés ces dernières années en termes de prise en charge thérapeutique avec des nouvelles molécules. Le pronostic fœto-maternelle est nettement amélioré par la prise en charge multidisciplinaire, la poursuite des traitements et la programmation de la grossesse loin des poussées.

### Etat des connaissances actuelle sur le sujet

- Les changements hormonaux dus à la grossesse présentent un impact sur l’évolution des maladies auto-immunes retentissant ainsi sur le déroulement et l'issue de la gestation;
- L'atteinte rénale, pulmonaire et la maladie thromboembolique artérielle et veineuse conditionnent le pronostic vital de la femme et engendrent la perte fœtale dans la majorité des cas;
- L’élaboration de plusieurs produits pharmaceutique a contribué à améliorer et espacer les rechutes des maladies auto-immunes.

### Contribution de notre étude à la connaissance

- Le suivi rapproché des gestantes portant une maladie auto-immune permet de détecter la période ou la patiente est plus vulnérable pour développer une rechute fonction du type de la maladie auto-immune;
- L'arrêt du traitement au cours de la grossesse est source d'apparition de rechute, d'où l'intérêt d'adapter une thérapeutique autorisée pendant la gestation;
- La prise en charge multidisciplinaire impliquant plusieurs spécialistes selon l'organe atteint aboutit un meilleur résultat.
